# Long-term population persistence of flightless weevils (*Eurhoptus pyriformis*) across old- and second-growth forests patches in southern Appalachia

**DOI:** 10.1186/s12862-018-1278-y

**Published:** 2018-11-09

**Authors:** Michael S. Caterino, Shelley S. Langton-Myers

**Affiliations:** 1 Department of Plant and Environmental Sciences, 277 Poole Agricultural Center, Clemson University, Clemson, SC 29634-0310 USA; 2 Ecoquest Education Foundation, Whakatiwai, Pokeno, New Zealand

**Keywords:** Appalachian Mountains, Phylogeography, Old-growth forest, Biodiversity, Weevils

## Abstract

**Background:**

Southern Appalachian forests are dominated by second-growth vegetation following decades of intensive forestry and agricultural use, although some old-growth patches remain. While it’s been shown that second-growth areas may exhibit comparable species richness to old-growth in the area, the extent to which populations of arthropods in second-growth areas have persisted vs. recolonized from other areas remains unexamined. The implications for conservation of both classes of forest are significant. Here we analyze population diversity and relatedness across five old-growth and five second-growth populations of flightless, leaf litter-inhabiting beetles in the genus *Eurhoptus* (Coleoptera: Curculionidae: Cryptorhynchinae). Our main goal is asking whether second-growth areas show diminished diversity and/or signals of recolonization from old-growth sources.

**Results:**

Population genetic and phylogenetic analyses do not reveal any consistent differences in diversity between the old-growth and second-growth populations examined. Some second-growth populations retain substantial genetic diversity, while some old-growth populations appear relatively depauperate. There is no phylogenetic indication that second-growth populations have recolonized from old-growth source populations.

**Conclusions:**

Most populations contain substantial and unique genetic diversity indicating long-term persistence in the majority of sites. The results support substantial resilience in second-growth populations, though the geographic scale of sampling may have hindered detection of recolonization patterns. Broad scale phylogeographic patterns reveal a deep break across the French Broad River basin, as has been reported in several other taxa of limited dispersal abilities. In *Eurhoptus* this break dates to ~ 2–6 Ma ago, on the older end of the range of previously estimated dates.

**Electronic supplementary material:**

The online version of this article (10.1186/s12862-018-1278-y) contains supplementary material, which is available to authorized users.

## Background

The southern Appalachian Mountains of eastern North America are home to an incredibly rich biota [1–4]. These mountains have been exposed and unglaciated for over 100 million years [5], much of their flora and fauna is ancient, and many groups have diversified extensively (e.g. amphibians reach their global peak diversity here [6]). Forests of the Appalachian Mountains also show a long history of human impacts, from pre-Columbian times through the present: small- to large-scale agriculture has converted many valleys, while timber harvesting extended to the summits of many mountains [7]. Indirect effects from invasive insect species like the balsam and hemlock woolly adelgids (*Adelges piceae* and *A. tsugae*, respectively), and diseases like Chestnut blight (the fungus *Cryphonectria parasitica*) have further altered Appalachian forests and impacted associated animal communities.

While the majority of Appalachian forests have been heavily impacted by anthropogenic pressures, patches of old-growth forests that have escaped at least some of these effects remain [8]. The largest tracts (~ 175,000 acres) are found in Great Smoky Mountains National Park, with another ~ 80,000 acres scattered in other protected areas (National Forests, primarily; [8]). There has been little work on how well these smaller old-growth fragments preserve unique native diversity, particularly with respect to their arthropod fauna. Some studies of the flora and the vertebrate fauna have concluded that old-growth forests preserve unique elements that have not persisted in secondary forests [9–13], though studies of insects have not consistently agreed. Pollinators seem to have benefitted from the relatively open canopies of logged forests [14]. Hunting spiders seem similarly to benefit from more open canopies and forest floors, whereas other more sedentary guilds of spiders disappeared following clearcutting [15]. In northern Appalachia, Chandler & colleagues [16, 17] compared species richness of litter-inhabiting beetles between old-growth and ~ 40 year old fragments, finding that beyond disappearance of a few old-growth specialists, overall species richness in second-growth forests was not seriously impacted. Examining the litter fauna associated with coarse woody debris (CWD) in southern Appalachia, Ferro et al. [18] found significantly lower species richness in primary forest litter than in secondary litter, though for species associated primarily with CWD, this was reversed. Our own work in this area has focused on leaf litter inhabiting beetles, a diverse community performing important roles in decomposition and nutrient cycling [19, 20]. We recently examined similarity in species composition across the litter beetle communities from a network of old-growth and second-growth sites, scattered across western North Carolina and upstate South Carolina [21]; second-growth communities are comparable in species richness, as well as in complementarity, with unique species being found at all second-growth sites.

As with our own previous work, essentially all studies that have compared arthropod diversity across old- and second growth forests have focused on the community level. But finding that comparable community-level species richness between old- and second growth forests may have competing explanations: either extinction following forest clearing is rare, or extirpated species (or others) are capable of recolonizing and finding suitable microhabitats in secondary forests. Studies at the community level cannot clearly distinguish between these possibilities. Yet the difference for conservation planning is highly significant. If species can persist in second-growth fragments, then those areas and their native populations should be the direct focus of conservation activities. Alternatively, if second-growth fragments owe their fauna to recolonization from better-preserved source areas, management efforts should focus more squarely on the old-growth reservoirs and on the corridors connecting them to recovering areas. Recent landscape-scale analyses have also emphasized the importance of improving corridors to facilitate species and community response in the context of climate change; many southern Appalachian forest fragments exhibit a critical level of isolation [22].

Distinguishing persistence from recolonization may be observed directly for larger plants and animals, whose populations may be relatively easily censused during recovery through visual surveys. However, for more cryptic elements of the biota, we must rely on indirect approaches. One especially valuable approach utilizes phylogeographic and population genetic analyses of molecular markers to reveal population-level relatedness, past demographic trends, and dispersal patterns. In salamanders, this approach has revealed regular correspondence between population-level diversity and forest age [23], supporting a recolonization scenario. Intraspecific molecular analysis can also reveal demographic effects in populations, such as bottlenecks and founder effects [24]. Here we carry out a detailed analysis of variation among southern Appalachian populations of a flightless, litter-inhabiting weevil, *Eurhoptus pyriformis* LeConte, hoping to shed more light on these questions.

*Eurhoptus* is a diverse genus of Curculionidae: Cryptorhynchinae, distributed throughout the eastern and central United States, and south into Central America [25]. While the diverse neotropical fauna remains largely undescribed, the U.S. species were recently revised to include eight species [26]. All *Eurhoptus* species are flightless and found in leaf litter, where they are presumed to feed on woody detritus, like many other litter-inhabiting Cryptorhynchinae [26, 27]. Related weevils (other flightless genera in the subfamily Cryptorhynchinae) have been shown to be good indicators of intact old-growth forest in Central Europe [28]. *Eurhoptus pyriformis* is the most widespread *Eurhoptus* species in the U.S. occurring from Georgia to Pennsylvania, and west to Illinois and Arkansas. The species exhibits considerable variation across this range, especially in scale patterning of the elytra. The exact distributions of these variants are not yet fully resolved, but both patterned and unpatterned forms are found in the southern Appalachians, and their relationships and distributions may be useful to resolving biogeographic history in the region.

Our primary goal is to evaluate recent population-level effects across patches of relatively undisturbed ‘old-growth’ and secondary growth stands, specifically asking the following questions:

1. Is genetic diversity greater in populations occupying old-growth stands of Appalachian forest than in nearby secondary growth?

Rationale: Past deforestation is expected to have had detrimental effects on the fauna of the leaf litter. Whether through imposition of a population bottleneck via reduced population size, or through local extirpation and recolonization (founder effects), the gross population genetic effects should be reflected by significantly reduced population level diversity.

2. In recovered, secondary forests where *Eurhoptus* populations presently exist, can we determine whether presence is due to persistence or recolonization?

Rationale: Phylogenetic relationships among populations should reflect comparative phylogeographic expectations. If populations have persisted through past deforestation, genetic markers should represent some subset of what was originally present, with reduced diversity depending on the severity of impact. If populations in secondary forests represent recolonization from less impacted areas, genetic markers should indicate closer than expected relationships to these source populations.

3. Do nuclear markers reveal comparable levels of population-level differentiation and similar broad-scale phylogeographic relationships to mitochondrial DNA?

Rationale: Mitochondrial markers alone tend to exaggerate population level divergences due to smaller effective population sizes. Rapidly evolving nuclear markers should yield more reliable estimates of population divergence, isolation, and relationships.

Additionally, these population level analyses will give us further insight into degree of differentiation among morphs of *Eurhoptus pyriformis*. Anderson & Caterino [26] considered the patterned and nonpatterned morphs to be conspecific, finding the patterned populations to be more broadly distributed than, and paraphyletic with respect to, the non-patterned populations. However, more detailed population level analyses will help resolve their fine-scale relationships.

## Results

### Population level diversity comparisons

**Fig. 1:**
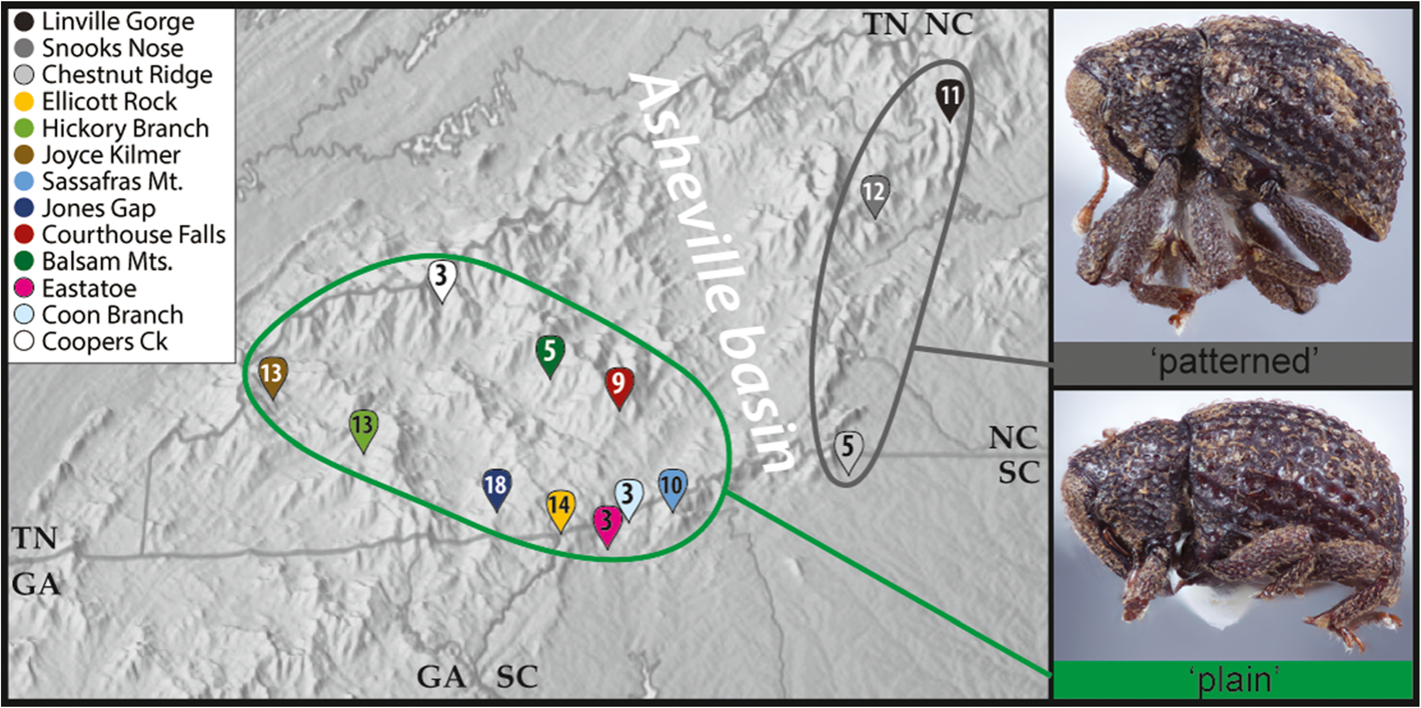
Map of the southern Appalachian Mountains displaying sites where *Eurhoptus* were collected between January 2015 to July 2016*.* Displayed within the balloons are the number of individuals sequences (for at least one gene) for each site. Colors of balloons represent the site, which are used for the corresponding network and tree figures. Inset photos represent specimens of the patterned and non-patterned morphs, and where they are located is indicated in green (plain) and black (patterned). Map adapted from www.shadedrelief.com, used with permission

Ten populations were sufficiently well sampled to include in population diversity analyses (see map in Fig. 1). These core populations included 110 individuals, 82 from the ‘plain’ morph and 28 of the patterned morph. Each collecting locality contained a single morph: The ‘plain’ morph was located in three old-growth sites, and four second-growth sites, while the patterned morph was found in two old growth sites and one second growth (Table 1). Cytochrome oxidase I sequences were available from 104 individuals of *E. pyriformis*, KKV from 54 (with 28 distinct alleles), and CAD from 48 (with 64 distinct alleles). ITS2 was represented by 49 sequences. Due to length variation, ITS2 sequences were difficult to phase, and we did not analyze this marker separately.

**Table 1:** Collection information of *Eurhoptus* specimens obtained from January 2015 to July 2016 for 10 sites across the southern Appalachian Mountains

Population (morph)	Status	Approx. lat/long	Elev. range (m)	COI seqs	CAD seqs	KKV seqs	ITS seqs
Included in diversity analyses				74	24	25	25
Linville Gorge (patterned)	Old Growth	35.94°N, 81.93°W	900–1100	9	7	10	6
Hickory Branch (plain)	Old Growth	35.22°N, 83.70°W	1050–1270	13	8	10	10
Snooks Nose (patterned)	Old Growth	35.72°N, 82.21°W	610–1050	12	8	8	8
Ellicott Rock (plain)	Old Growth	34.98°N, 83.08°W	670–860	14	4	5	5
Joyce Kilmer (plain)	Old Growth	35.34°N, 83.97°W	820–900	13	2	1	4
Balsam Mts (plain)	2nd Growth	35.35°N, 83.11°W	1000–1640	5	4	4	2
Chestnut Ridge (patterned)	2nd Growth	35.14°N, 82.28°W	330–450	5	2	3	3
Jones Gap (plain)	2nd Growth	35.07°N, 83.28°W	1280–1360	18	5	5	5
Courthouse Falls (plain)	2nd Growth	35.27°N, 82.89°W	1030–1050	9	4	4	4
Sassafras Mt. (plain)	2nd Growth	35.06°N, 82.77°W	1040–1070	10	3	3	2
Included only in phylogenetic analyses							
Coon Branch (plain)	Old Growth	35.03°N, 83.01°W	610	3	2	2	2
Cooper’s Ck (plain)	2nd Growth	35.48°N, 83.38°W	700–750	3	1	1	1
Eastatoe Preserve (plain)	mixed	35.04°N, 82.81°W	430	3	3	3	3
GA: Walker Co.	2nd Growth	34.93, 85.37	340	1		1	
SC: Pee-Dee	2nd Growth	34.39, 79.71	18	2		1	
SC: Oconee	2nd Growth	34.74, 83.18	267	1			
Arkansas	2nd Growth	35.17, 93.64	770	1		1	
Indiana	2nd Growth	40.4, 86.9	175	1			
Outgroups (NC, SC, IL, IN, KY, AL, AR, TX)				30	6	24	25

Top half shows all locations included in population level analyses. Below that are individuals added only for phylogenetic analyses. Outgroups include three other species of *Eurhoptus* (*E. curtus*, *E. sordidus*, and *E. aenigmaticus*) as well as two species of *Acalles* and one species of *Peracalles*

**Fig. 2:**
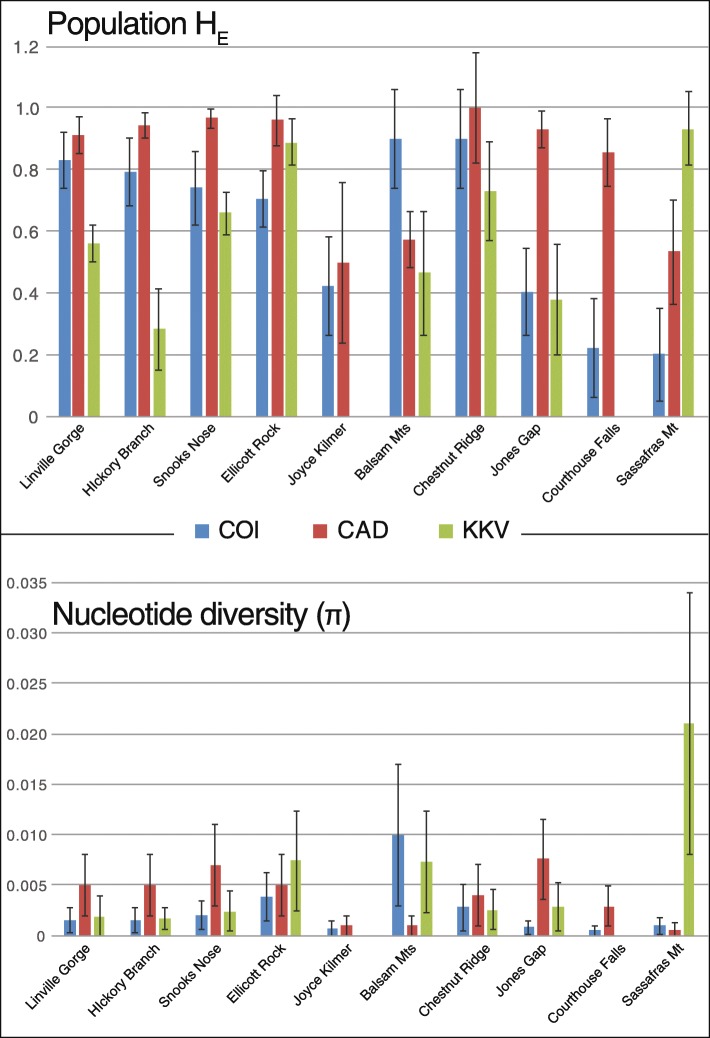
Comparisons of *Eurhoptus* population diversity statistics between sites. Three genes are displayed COI (blue), CAD (red) and KKV (green). The top graph shows population heterozygosity with standard error. The bottom graph shows nucleotide diversity with standard error

There was no significant pattern of population level diversity differences in old-growth versus second-growth populations, as assessed by either haplotype diversity or nucleotide diversity (Table 2 and Fig. 2). Haplotype diversity in mitochondrial DNA was generally moderate to high, and no population was monomorphic for the individuals analyzed. Mean mitochondrial haplotype diversity in old-growth populations exceeded that in second-growth (0.698 vs. 0.525), but this difference was not significant (*p* = 0.7). Noteably, the two populations with the highest mitochondrial haplotype diversity (Balsam Mts. and Chestnut Ridge, both 0.90 ± 0.16) both represented second-growth areas. The lowest mitochondrial haplotype diversities were also found at two second-growth sites (Sassafras Mt., 0.20 ± 0.15, and Courthouse Falls/BRP, 0.22 ± 0.16), but that of the old-growth Joyce Kilmer population was only slightly higher, at 0.42 ± 0.16.

**Table 2:** Basic population genetic statistics of *Eurhoptus* populations sampled throughout the southern Appalachians

Population	Status	mt	mt	mt	mt	CAD	CAD	KKV	KKV
		H_*E*_	훑	Tajima D	Fu’s Fs	H_*E*_	훑	H_*E*_	훑
Linville Gorge	Old Growth	0.83 ± 0.09	0.002 ± 0.001	−0.689	**−1.99**	0.91 ± 0.06	0.005 ± 0.003	0.56 ± 0.06	0.002 ± 0.002
Hickory Branch	Old Growth	0.80 ± 0.11	0.002 ± 0.001	−1.44	**−4.12**	0.94 ± 0.04	0.005 ± 0.003	0.28 ± 0.13	0.002 ± 0.001
Snooks Nose	Old Growth	0.74 ± 0.12	0.002 ± 0.001	0.09	−0.61	0.97 ± 0.03	0.007 ± 0.004	0.66 ± 0.07	0.002 ± 0.002
Ellicott Rock	Old Growth	0.70 ± 0.09	0.004 ± 0.002	1.505	2.45	0.96 ± 0.08	0.005 ± 0.003	0.89 ± 0.08	0.007 ± 0.005
Joyce Kilmer	Old Growth	0.42 ± 0.16	0.001 ± 0.001	−1.77	**−1.56**	0.50 ± 0.26	0.001 ± 0.001	0.00 ± 0.00	0.000 ± 0.000
Balsam Mts	2nd Growth	0.90 ± 0.16	0.010 ± 0.007	−1.23	1.24	0.57 ± 0.09	0.001 ± 0.001	0.46 ± 0.20	0.007 ± 0.005
Chestnut Ridge	2nd Growth	0.90 ± 0.16	0.003 ± 0.002	0.699	−1.40	1.00 ± 0.18	0.004 ± 0.003	0.73 ± 0.16	0.003 ± 0.002
Jones Gap	2nd Growth	0.41 ± 0.14	0.001 ± 0.001	−2.03	**−2.32**	0.93 ± 0.06	0.008 ± 0.004	0.38 ± 0.18	0.003 ± 0.002
Courthouse Falls	2nd Growth	0.22 ± 0.16	0.001 ± 0.001	1.36	0.67	0.86 ± 0.11	0.003 ± 0.002	0.00 ± 0.00	0.000 ± 0.000
Sassafras Mt	2nd Growth	0.20 ± 0.15	0.001 ± 0.001	−1.66	**1.74**	0.53 ± 0.17	0.001 ± 0.001	0.93 ± 0.12	0.021 ± 0.013
	**OG Avg**	**0.70**	**0.0019**			**0.86**	**0.0046**	**0.48**	**0.0027**
	**SG Avg**	**0.53**	**0.0030**			**0.78**	**0.0032**	**0.50**	**0.0067**

Bold Fu’s F_s_ are significant

Nucleotide diversities for mitochondrial haplotypes were generally low, with most haplotypes within populations differing by one or two mutational steps. Old-growth and second-growth mitochondrial nucleotide diversities were not significantly different. Nucleotide diversities for haplotypes followed similar patterns to haplotype diversities, with similar levels across most old-growth sites, though distinctly lower at Joyce Kilmer (avg. 0.00194). The high and low extremes were both found in second-growth sites (0.01 in Balsams and 0.0005 at Courthouse Falls, respectively).

Similarly, nuclear gene diversities (*H*) do not reveal simple old-growth vs. second-growth differences. CAD’s higher average gene diversity at old-growth sites (0.856 vs. 0.779; see Fig. 2) is not significant (*p* = 0.57). It is again lower than average for the old-growth Joyce Kilmer site (0.50), and shows a wider range across second-growth sites. KKV gene diversities were more variable across all sites, with insignificantly different means across old- and second-growth (0.478 vs. 0.501; *p* = 0.92), and the highest value at the second-growth Sassafras Mt. site (0.933 ± 0.12). Nevertheless, nuclear gene sampling was quite low for some populations, making some of those cross-gene comparisons tentative. Nucleotide diversity differences between old- and second growth populations were not significantly different for either CAD or KKV (*p* = 0.41 and *p* = 0.33, respectively).

**Fig. 3:**
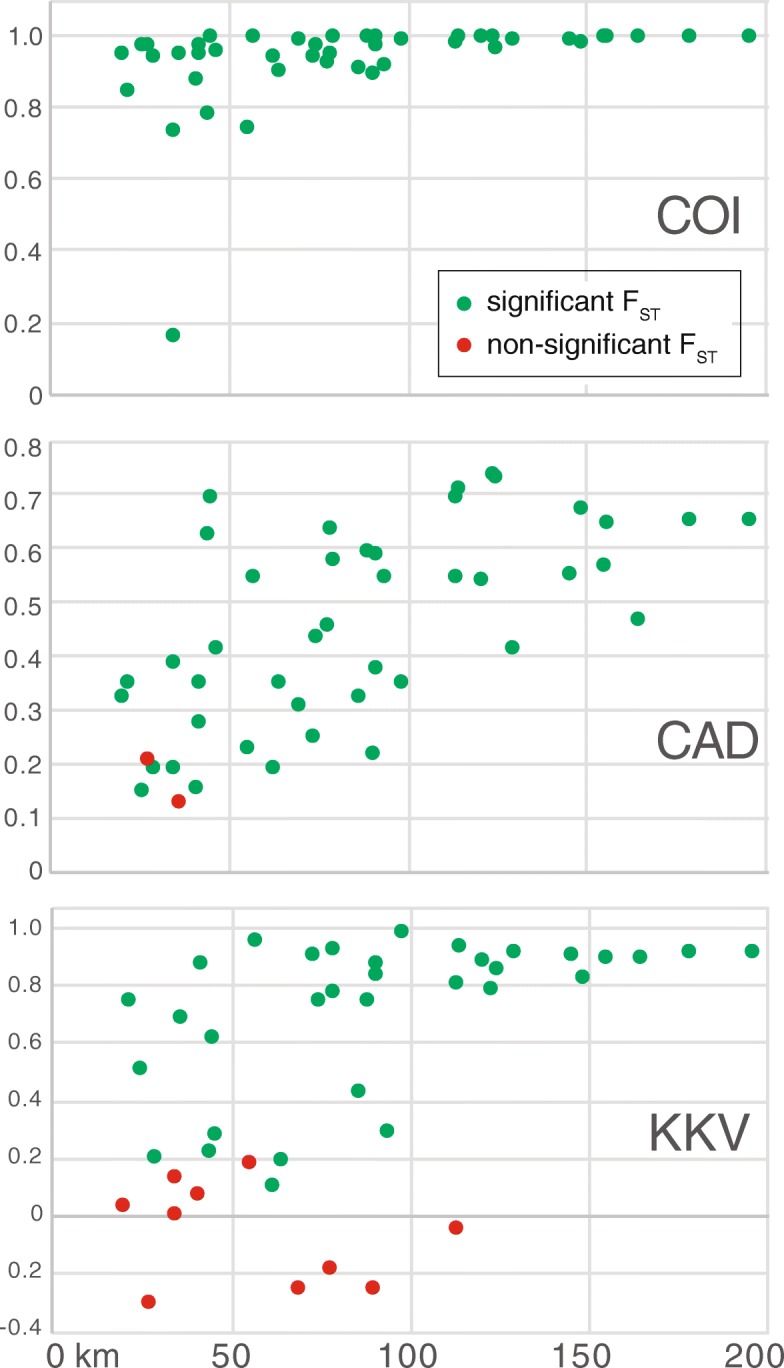
Variation of F_ST_ associated with the straight-line distance (km) between populations. F_ST_ for each individual pairwise population comparison is displayed for each gene. Non-significant F_ST_ values are indicated in red

Populations showed low connectivity according to F_ST_ values for mitochondrial data (Fig. 3); all pairwise comparisons were highly significant. The lowest F_ST_ value was 0.15 between Linville Gorge and Snooks Nose patterned populations. All others exceeded 0.7. Most comparisons for the more slowly evolving, diploid nuclear markers were also significant, though with a few exceptions. In CAD there were two non-significant pairs: Ellicott Rock to Courthouse Falls, and Hickory Branch to Joyce Kilmer. The first of these may be pertinent to our recolonization question, in that Ellicott Rock is an old-growth site somewhat near (~ 35 km) the second-growth Courthouse Falls population. The other non-significant result is between two old-growth sites. In the least divergent gene, KKV, numerous comparisons were non-significant, including many pairs of western populations (especially Hickory Branch, Joyce Kilmer, Jones Gap, and Balsam Mts; Ellicott Rock with the last three of these), and the Linville Gorge-Snooks Nose pair of northeastern populations. Mantel tests on F_ST_ vs. straightline distances between populations were all highly significant (*p* < 0.003), indicating strong isolation by distance (Fig. 3).

Several populations exhibited negative or significantly negative values for Tajima’s D or Fu’s F_S_, respectively (Table 2), based on mitochondrial data (none were significant for CAD or KKV). Negative values indicate an excess of rare alleles, and are commonly taken to indicate recent population expansion (such as following a bottleneck, as would be most relevant to the current study; 39). Most such results are observed in the old-growth populations sampled (Linville Gorge, Hickory Branch, and Joyce Kilmer), with Jones Gap showing the only significantly negative result for Fu’s F_S_ among second-growth populations. It may be that this indicates some level of impact to all populations in the region, with the lag to population recovery being greater in second-growth populations.

### Individual gene phylogenies

**Fig. 4:**
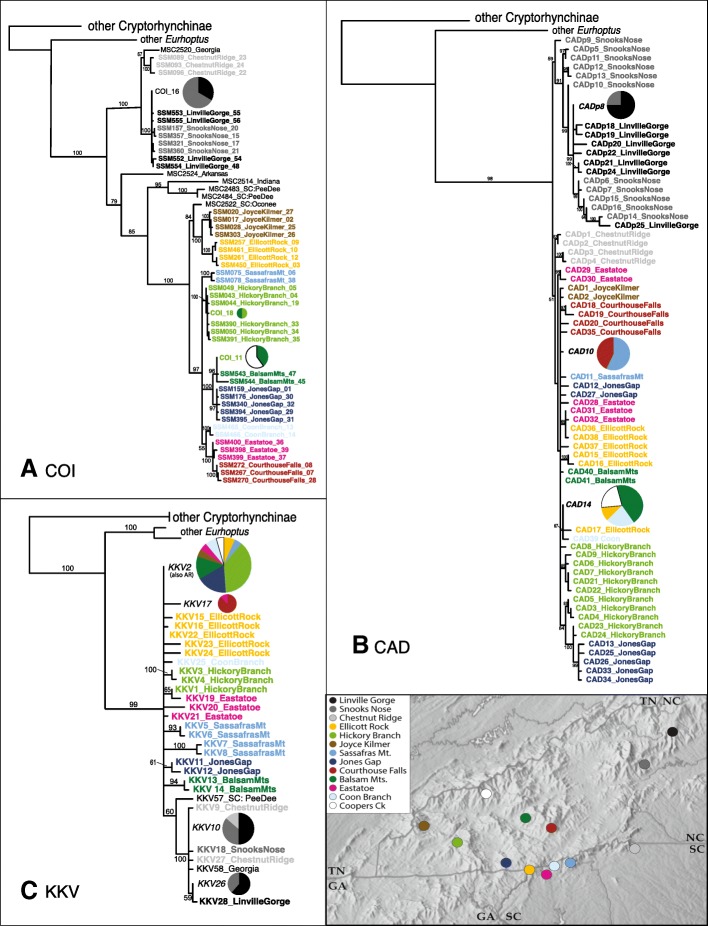
Bayesian phylogenies for *Eurhoptus* specimens based on COI (**a**), CAD (**b**) and KKV (**c**) gene haplotypes. Each phylogeny only includes unique haplotypes. Posterior probabilities are used as a measure of support and are labelled above the branches. Names of taxa include a haplotype/ allele designation and site name; for COI, a representative individual extraction number precedes these. Widespread alleles are indicated using pie charts showing representation across sites (keyed to colors in map inset). Map adapted from www.shadedrelief.com, used with permission

**Fig. 5:**
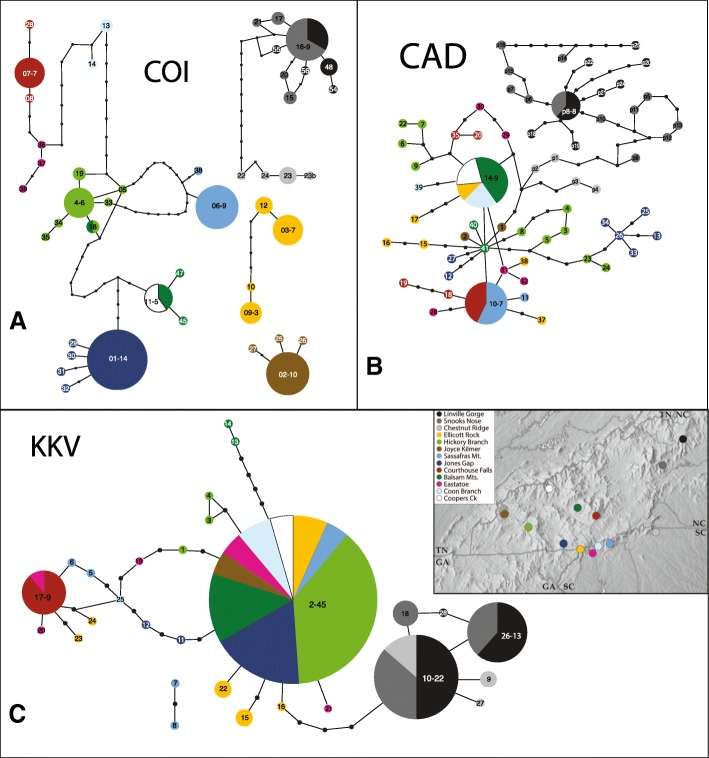
Statistical parsimony networks for *Eurhoptus* based on COI (**a**), CAD (**b**), and KKV (**c**) haplotypes/alleles for all individuals. Haplotype numbers for each gene match those in Fig. 4 Bayesian phylogenies. The colour of the circle and pie-charts correspond to the locality of origin (see map insert). The size of the circles is proportionate to the number of individuals with that haplotype (COI) or to the total number of copies of that allele (CAD and KKV, both based on phased sequences) in each population. In larger circles, the number of copies is indicated following the haplotype number (e.g. 01–14). Small black circles represent inferred intermediate haplotypes. Map adapted from www.shadedrelief.com, used with permission

Phylogenies of alleles reveal more subtle patterns of interpopulational connectedness than the diversity statistics do. For COI, most populations represent monophyletic (Fig. 4a) and endemic groups of haplotypes. One exception is that the old-growth Linville Gorge and Snooks Nose populations shared a dominant haplotype (#16), with several other haplotypes in these populations differing from it by three or fewer mutations (Fig. 5a). These localities are about 30 km apart. Also, the Balsam Mts. share a haplotype (#11) with the Coopers Creek population (both considered second growth). These are also about 30 km apart. Lastly (for COI), one haplotype is shared between the Balsam Mts. and the old-growth Hickory Branch population, almost 60 km away. Nuclear allele relationships display minimal geographic signal compared with mtDNA. Though numbers of alleles are comparable to those for mtDNA (allowing for sampling differences), divergences are shallower in both CAD and, especially, KKV. There is also considerably more sharing of alleles among populations (Figs. 4b-c, 5b-c). No second growth-site is both depauperate in alleles and derived from within a nearby old-growth site.

### Broader patterns of phylogenetic relationship across *E. pyriformis*

**Fig. 6:**
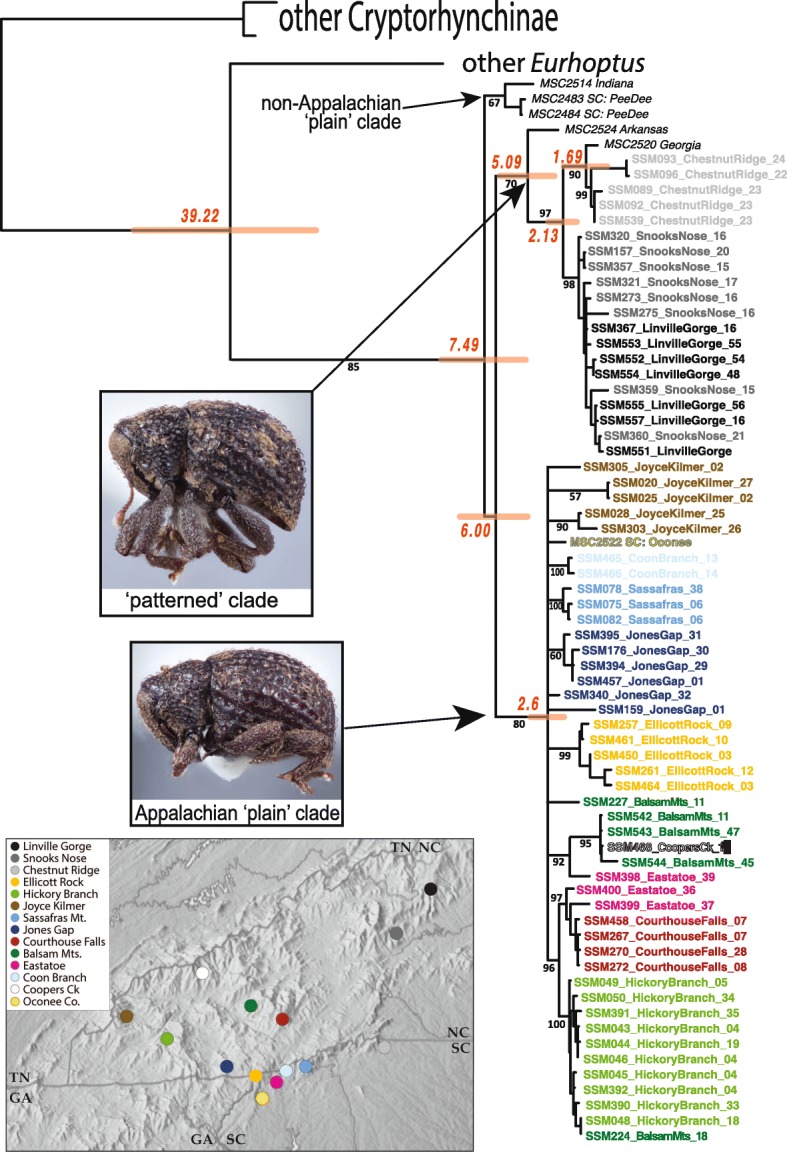
Bayesian phylogeny for *Eurhoptus* specimens based on the combined dataset of COI, CAD, KKV, and ITS sequences. This includes all unique individuals (small numbers of individuals identical for all genes were removed), with non-focal populations and taxa removed. Name of taxa includes: a representative extraction number, locality, and COI haplotype number. Numbers on branches indicate posterior probabilities. Colors are keyed to localities as shown in map inset. Inset photos represent specimens of the patterned and non-patterned morphs. Map adapted from www.shadedrelief.com, used with permission

Combined data phylogenetic analysis (Fig. 6) does not conclusively resolve all plain and patterned lineages. A small lineage of extralimital (Indiana + coastal South Carolina) ‘plain’ populations forms a sister group to patterned and plain clades comprising the Appalachian populations, but with only weak support (*p* = 0.61). Within these main clades, these morphs show only a single shared allele in any gene (one patterned individual from Arkansas shares the widespread KKV2 allele). The COI phylogeny (Fig. 4a) resolves patterned morphs as a paraphyletic group with respect to plain populations. CAD resolves Appalachian patterned populations as paraphyletic with respect to plain. The Chestnut Ridge patterned population is particularly unstable: Combined data (and COI alone) resolve it (along with a single individual from northwest Georgia) as sister to the patterned Snooks Nose plus Linville Gorge lineage. In CAD, on the other hand, Chestnut Ridge is monophyletic with plain populations. In KKV Chestnut Ridge is indistinguishable from Snooks Nose and Linville Gorge populations, sharing alleles with both. Non-patterned populations exhibit few deep subdivisions. Larger clades seemingly well-supported by COI (e.g. Balsam Mts + Jones Gap, Joyce Kilmer + Ellicott Rock) do not hold up in combined analysis. Most populations remain monophyletic, but there is little support for any closer relationships between any of them.

BEAST analysis dated the deepest divergences within *E. pyriformis* to 7.5 Ma ago. The more widespread and apparently ancestral patterned form gave rise to the plain clade (which is restricted to mountainous areas west of the Asheville Basin) as much as 6 Ma ago. Separate diversification within western and eastern sets of populations has proceeded over more than 2 Ma.

## Discussion

Our analyses did not reveal any significant difference in population-level diversity in second-growth as compared with old-growth forests. Although some second-growth populations did seem to have depressed levels of COI variation (Courthouse Falls, Sassafras Mt.), population by population results were more individual, with a wide range of variation in diversity measures in either gross ‘forest history’ category. Some old-growth areas were revealed to be low in genetic diversity, such as Joyce Kilmer, which exhibited very low haplotype and nucleotide diversity across all markers. On the other hand, in at least some markers, some second growth areas exhibited high diversity, with the highest COI diversity found in the Balsam Mts., the highest KKV diversity at Sassafras Mt., and the highest CAD diversity at Jones Gap. Apart from the Joyce Kilmer outlier, old-growth populations showed lower variability than second-growth populations did across most measures, and we suggest that this reflects the variety of impacts that second-growth populations may have experienced. This would be consistent with observations that forest clearing creates some novel microhabitats [14, 15], and that some resident organisms may benefit at intermediate levels of disturbance [29, 30]. This may be unlikely to explain the high nucleotide diversities observed in only some markers. However, if we consider all these populations to be impacted to some degree (rather than looking at any as ‘pristine’), it is more reasonable to expect that direct impacts will not be uniform, or that higher variability reflects a chaotic or unpredictable response. Testing this would require more intensive and carefully structured sampling across areas with more detailed, known histories.

Our initial expectation that population level relationships might reflect post-disturbance recolonization appears to have been naïve. This prediction would only have been borne out by observations of limited (founder effect) diversity in recolonized populations, with close relationships to some better preserved population. We have not sampled all possible source populations, but the lack of a signal of founder effects in the second-growth populations precludes this explanation in any case. Further evidence against any repopulation scenario is found in the uniformly significant F_ST_ values (at least in COI) across all population comparisons. The diversity and monophyly of COI haplotypes in most populations indicates that their respective lineages have diversified in place for a significant portion of their histories. Contrasting significant COI F_ST_ values with some non-significant values in nuclear markers provides important insight into possible historical connections among populations. Several populations in the Blue Ridge region of North and South Carolina share nuclear alleles. The Snooks Nose and Linville Gorge populations of the patterned morph also show non-significant F_ST_ values in KKV, and they share haplotypes/alleles of both KKV and COI markers. Two CAD alleles are widespread: allele CAD10 is shared by Sassafras Mt. and Courthouse Falls (both second-growth); allele CAD14 is shared by Balsam Mts. and Cooper’s Ck (both second-growth), as well as by Ellicott Rock and Coon Branch (both old-growth). The old-growth Hickory Branch population exhibits the greatest phylogenetic diversity of CAD alleles, with an endemic lineage and one from which Jones Gap (second-growth) alleles have arisen. Jones Gap also has two unrelated alleles and thus contains substantial phylogenetic diversity.

There is less overall allelic diversity in KKV, with very low divergence among alleles. No populations are both monophyletic and distinct from all others. In the patterned group two common alleles (KKV10, KKV26) span Snooks Nose and Linville Gorge old-growth areas, and KKV10 is present at the second-growth Chestnut Ridge site as well, which also has two unique single mutation derivatives. Each population has three total alleles, none differing by more than 2 mutational steps. One common allele occurs in all the plain populations except Courthouse Falls, where all individuals share a quite distinct allele (KKV17). All second-growth populations show a mix of common and unique alleles, with most (Jones Gap, Balsam Mts., and Sassafras Mt.) possessing divergent alleles and/or lineages. The last two of these exhibit KKV nucleotide diversities equaling or exceeding any old-growth population. Together, the combination of mitochondrial and nuclear markers simultaneously reveals significant modern-day isolation of most populations as well as historical patterns of connectedness.

At both deep and shallow phylogenetic levels, our results indicate an important biogeographic role for the Asheville/French Broad River basin, as many previous studies in this region have found [31–39]. Indeed this gap corresponds largely with our patterned and plain morphs, and the divide is not bridged by any haplotypes or alleles. Crespi et al. [36] have dated this gap to approximately 4.25 Ma ago in *Desmognathus* salamanders, while Browne & Ferree [32] suggest divergences across this gap might have been finalized (in red-backed voles) ~ 5000 years BP. The age of isolation of the plain *E. pyriformis* clade west of the Asheville basin appears intermediate between these, with common ancestors of the extant lineages dating to approximately 2 Ma ago (though the deepest split between them may have been as long as 6 Ma ago). The apparent inclusion of some extralimital representatives in the northeastern clade, however, prompts caution in this interpretation. More comprehensive geographic sampling will be necessary to clearly hypothesize where and when these divergences may have arisen.

With regard to the status of patterned and plain morphs of *E. pyriformis,* the broader phylogeny is not sufficiently resolved (or represented) to provide more light on their respective monophylies. Both morphs extend beyond our focal region to areas where our sampling is limited. Our combined phylogeny weakly suggests plain morph paraphyly, with a widespread patterned clade derived from within. Nonetheless, the plain populations of southern Appalachia do represent their own well supported clade*.* Isolation of this lineage in a small area west of the Asheville basin rather unique. The exact limits of the different morphs around the southern and western margins of southern Appalachia beg to be further explored, as it seems that other less widely appreciated biogeographic barriers may be at work keeping this plain lineage isolated. Resolving the deeper history of this lineage will also require more intensive and broader geographic sampling.

Within morphs, population relationships are all very close, and geographical signal is minimal. Even in the relatively deeply divergent mitochondrial gene, where some population level relationships appear strongly supported, there is little clear correspondence with geography. Strongly supported COI clades like Joyce Kilmer + Ellicott Rock (100%PP), Balsam Mts + Coopers Creek + Jones Gap (100%PP), or Eastatoe + Courthouse Falls (100%PP) find no support in either the CAD or KKV trees. None has obvious geographic correspondence either, suggesting that random coalescence of ancestral COI polymorphism may have resulted in spurious phylogenetic signal. Some subtler signal may yet emerge with more intensive population level sampling (capturing a greater diversity of rare alleles).

## Conclusions

This study amplifies surprising conclusions from our previous work on species richness across these old- and second-growth communities [21]: the stands identified as second-growth appear to host diverse native populations (and communities). They do not exhibit any consistent sign of severe past population declines. While there are exceptions, with certain markers appearing depauperate in some populations, this is also true of some of the old-growth populations included. Nearly all populations are polymorphic at most loci, which largely precludes our inferring any extirpations followed by recolonization. The most reasonable explanation of these findings is that most populations have persisted through the past three centuries of increasingly intensive forest use, and that such impacts have not been so severe as to completely extirpate populations in the areas sampled. We acknowledge some risk of sampling bias here, in that localities where we have not been successful in sampling *Eurhoptus* have of course not been included. The historical record of *Eurhoptus* collection is very sparse, so we have very little basis for knowing where they should and should not be found, or evidence for populations in areas where they cannot now be found. Permanent extirpations cannot be ruled out. As we continue to sample across this area, and continue to develop a clearer picture of diversity patterns, we hope that our findings continue to indicate that humans’ impacts on southern Appalachian forests have not been severe or irreversible.

## Methods

### Sampling sites

Our core sampling involved gathering *Eurhoptus* specimens from 10 main sites within protected areas of western North Carolina and northwestern South Carolina. Five of these sites are considered to be old-growth (following [8]), meaning that they have not been systematically logged in the past (though all have experienced some anthropogenic impacts, discussed further below). The other five represent secondary forest that has been substantially logged at some time in the past, and these now largely comprise regrowth. In addition to these southern Appalachian sites, we included other *Eurhoptus* specimens selected from additional areas and species to provide phylogenetic context for the ‘ingroup’. Basic site information and numbers of samples included is summarized in Table 1, and their locations are shown in Fig. 1. All specimens used and their sampling sites are listed in Additional file 1.

The old-growth sites are: *The Ellicott Rock Wilderness,* containing 185 acres of old-growth extending across Cherokee National Forest in Georgia, Sumter National Forest in South Carolina, and Nantahala National Forest in North Carolina. We sampled only within North and South Carolina. Both sampling sites within Ellicott Rock Wilderness are relatively low in elevation (< 1000 m), and mainly comprise hardwood and hemlock forest along the Chattooga River and some of its tributaries. *Joyce Kilmer Wilderness*, in the Nantahala National Forest, includes 5962 acres of old-growth forest, and is similar in composition and elevational range to Ellicott Rock. We sampled from a relatively small area along Santeetlah Creek. The *Hickory Branch* area is an oak-hickory forest, also within Nantahala National Forest. Specific sampling sites range from about 1000–1300 m. The *Snooks Nose* area represents the largest old-growth tract within Pisgah National Forest. Our samples come from lower to mid-elevations (600–1050 m) which are mainly oak, with hemlock in the coves, and some pine at the higher elevations. *Linville Gorge Wilderness*, part of the Pisgah National Forest, includes large areas of unlogged old-growth, due to its steep sides and inaccessibility. Our sampling sites were on the west side of the gorge between 900 and 1100 m.

The second-growth sites samples were obtained from: *Chestnut Ridge Heritage Preserve*, managed by the South Carolina Department of Natural Resources. This site includes a range of early successional forest to mature cove hardwoods. The *Jones Gap* site follows the Bartram Trail, in the Nantahala National Forest, and comprises mainly hardwood forest with young patchy undergrowth. The *Balsam Mountains* site lies within a private preserve, formerly paper company land, that was historically logged for pulp. It includes rich cove forests, drier oak-hickory stands, northern hardwood, and high elevation red oak. *Courthouse Falls*, in Pisgah National Forest, is similar in composition, and rather close (~ 20 km) to the Balsam Mountains site. These samples ranged from lower cove forests around 1000 m to higher oak-hickory forest near the Blue Ridge Parkway on the western side of the canyon around 1400 m. Lastly, *Sassafras Mountain* is the highest peak in South Carolina. While the summit has been cleared largely of dead hemlocks (*Tsuga* sp.), the nearby slopes have well developed secondary hardwood forest with considerable *Rhododendron* shrub cover.

### Specimens

We collected weevil specimens by sifting leaf litter. For each sample we used an 8 mm mesh to sift approximately 2 m^2^ of leaf litter from the forest floor. We bagged the sifted portion and brought the sample back to the lab where we placed it into Berlese funnels that extracted the live invertebrates into 100% ethanol. We attempted to collect a minimum of ten individuals from each sampling locality (from multiple specific samples). We used a total of 125 *Eurhoptus pyriformis* specimens for analysis. These include four from outside the Appalachian region (two from the coastal plain of South Carolina, one from Indiana, and one from Arkansas), excluded from population level analyses. Two singleton specimens from Appalachian Georgia and upstate South Carolina localities were also excluded from population level analyses due to inadequate numbers. To provide phylogenetic context we included sequences from an additional 38 *Eurhoptus* specimens of other species (*E. curtus* Hamilton, *E. aenigmaticus* Anderson & Caterino, and *E. sordidus* LeConte), and an additional 6 specimens from outgroup genera *Peracalles* and *Acalles* (in the same subfamily Cryptorhynchinae). We obtained deeper outgroup sequences of three other Cryptorhynchinae from GenBank for two genes (from [27]). Voucher data for all included individuals is contained in Additional file 1. GenBank information for all distinct haplotypes or alleles is presented in Additional file 2.

### Molecular lab procedures

We dissected each specimen and used the GeneJet Genomic DNA Purification Kit (ThermoFisher Scientific, Waltham, MA) to extract DNA from the dissected head and prothorax. Following tissue digestion we removed the remaining exoskeleton and mounted the body parts as vouchers. All vouchers are deposited in the Clemson University Arthropod Collection. We targeted four genes for this study. Primers for these are provided in Table 3. We attempted to sequence 826 bases of the mitochondrial cytochrome oxidase I (COI) gene from all individuals (not all succeeded). COI is among the most widely used markers for intraspecific phylogeography in animals, and is almost universally variable within and among populations [40]. Assuming that nuclear genes would show lower levels of divergence, we subsampled among these for three nuclear markers, attempting to amplify at least one representative of each mitochondrial haplotype for each nuclear marker. We sequenced 1563 bases (as aligned with gaps) of the ITS2 ribosomal intergenic spacer from 75 individuals. ITS sequences have frequently been useful in resolving relationships among closely related populations in beetles (e.g. [41, 42]). We sequenced 921 bases of the protein-encoding gene CAD (*rudimentary*) from 64 individuals. Although CAD is more typically employed for deeper phylogenetic analyses (e.g. [43, 44]), it was recently used to help resolve intraspecific relationships in *Geodercodes* weevils [45]. Finally we sequenced 344 bases spanning an intron in the *krotzkopf verkehrt* (KKV) gene that encodes chitin synthase from 86 individuals. This fragment of KKV was shown to exhibit valuable intraspecific variation among western banded glowworm beetle populations [46]. Amplifications via PCR started with a 3 min initial denaturation at 95°, a 35–40 cycles of 30 s denaturation at 94°, 50–62° annealing for 30 s (see Table 3 for details), and a 1 min extension at 72°, followed by a 5 min final extension at 72°. Successful PCR products were sent to Macrogen USA (Rockville, MD) for Sanger sequencing in both directions. Sequence chromatograms were compiled, inspected, and preliminarily aligned in Geneious (Auckland, NZ). Length variable sequences (ITS2, CAD, and KKV) were aligned using the online version of MAFFT (v. 7; [47]), using the default settings (Strategy: Auto; ‘Try to align gappy regions anyway’, Scoring matrix ‘200PAM/k = 2’, Gap opening penalty: 1.53, Offset value = 0.0, nzero, Guide tree: default). The aligned, final, combined data set is available as Additional file 3.

**Table 3:** Oligonucleotide primer sequences and the conditions used for PCR amplification of genes amplified from *Eurhoptus* samples

Gene	Forward Primer	Reverse Primer	Anneal temp	Cycles	Reference
Cytochrome oxidase I	CAACATTTATTTTGATTTTTTGG (‘Jerry’)	TCCAATGCACTAATCTGCCATATTA (‘Pat’)	50	35	Simon et al., 1994
CAD	AGCACGAAAATHGGNAGYTCNATGAARAG (CD821F)	GCTATGTTGTTNGGNAGYTGDCCNCCCAT (CD1098R2)	53	37	Wild & Maddison 2008
kkv	TCGACCATHGCCAAYATCATGGA (KKV2768F)	GTACCNACDATNACNGCCATCAT (KKV3023R)	48	40	Polihronakis & Caterino, 2012
ITS2	GTTTCCGTAGGTGAACCTGC (TW81)	AATGTGCGTTCGAAATGTCG (HITR)	50	35	Richards et al., 1997

Analytical methods: To address our first question, whether genetic diversity in old-growth forest populations was significantly greater than those in secondary forest, we calculated gene diversity (*H*_*E*_) (or haplotype diversity, *h*, for COI) and nucleotide diversity (*π*) for each population, for each locus. These comparisons included 10 total populations (5 old-growth and 5 s-growth), representing the two morphologically distinct sets of populations discussed above. We calculated these diversity measures in Arlequin (v. 3.5; [48]), treating all populations separately. We compared haplotype diversity and nucleotide diversity across pooled old-growth and pooled second-growth sites using one-way ANOVA in JMP Pro (v. 13; SAS, Cary, NC). To assess possible connectivity among populations we calculated F_ST_ for each marker, incorporating Tamura-Nei distances among haplotypes/alleles. To evaluate the strength of isolation-by-distance relationships among populations we used Mantel tests (in Arlequin) to compare F_ST_ and straight-line distances calculated between sampling point centroids. To detect demographic trends we calculated Fu’s Fs [49] and Tajima’s D [50] for all populations using Arlequin.

In order to distinguish between the persistence and recolonization scenarios for those populations occurring in secondary forest, we used the results of a combined gene Bayesian phylogenetic analysis to ask the following questions about each such population (considering distinct species separately):

1. Are populations in second-growth areas reciprocally monophyletic with respect to nearest old-growth populations, or do they represent descendants of some nearby old-growth areas?
2. Do populations in second-growth areas contain appreciable phylogenetic diversity, or is reduced diversity consistent with one or few recent founders observed?

We carried out these analyses using MrBayes (v.3.2.6; [51]) on the CIPRES Science Gateway [52], using a partitioned four-gene data set represented by unique sequences only (no more than one individual identical across all available fragments was included). For combined analysis heterozygous nucleotide positions in CAD, KKV and ITS2 were represented by IUPAC ambiguity codes. The ideal partitioning scheme was determined by PartitionFinder using RaxML (v.2.1.1; [53, 54]), which resulted in 11 partitions (COI codon positions; ITS2, CAD coding sequence 1 codon positions, CAD intron, CAD coding sequence 2 codon positions, and KKV [largely intron]). The selected model for each partition is shown in Table 4. Other MrBayes parameters included: nruns = 2, ngen = 10,000,000, nchains = 4, burninfrac = 0.25. For comparison, we ran Bayesian analyses on individual gene data sets as well, under the same model parameters. Mitochondrial haplotypes were represented by one individual each. CAD and KKV alleles were distinguished using PHASE (as applied in DnaSP; [55]), and the Bayesian analyses were run on unique alleles only. We used the same settings as for combined data analyses.

**Table 4:** Genes, partitions, and nucleotide substitution models of *Eurhoptus* sequences

Gene	category	model
COI	codon position 1	GTR + G
COI	codon position 2	GTR + I + G
COI	codon position 3	GTR + G
CAD	cds1-codon position 1	K80
CAD	cds1-codon position 2	SYM + G
CAD	cds1-codon position 3	JC
CAD	intron	HKY
CAD	cds2-codon position 1	GTR + G
CAD	cds2-codon position 2	GTR + I + G
CAD	cds2-codon position 3	HKY + G
KKV	intron	HKY + G
ITS2	rDNA	HKY + G

We used BEAST (v. 1.10.0; [56]) to estimate a dated phylogeny for the combined data. We used COI and ITS2 rates estimated by Andujar et al. [57] for *Carabus* ground beetles. For COI we implemented their ‘cox1-b’ rate (corresponding to the fragment sequenced here) of 0.01–0.0198 substitutions/site/MY/lineage as a strict clock uniformly distributed prior. And for ITS2 we set 0.0035–0.0081 substitutions/site/MY/lineage as a uniform mean prior. A similar COI rate was used by [58] in the related flightless Australasian Cryptorhynchine weevil genus *Trigonopterus*, and was found to be consistent with geological calibrations. To enforce to the estimated rates, we implemented four partitions in BEAST corresponding to gene fragments (not partitioning by codon position), and input the result of our highly partitioned Bayesian analysis as a constraint (no rearrangements) tree. CAD and KKV partitions were assumed to have independent, unconstrained clocks. BEAST simulations were run for 10,000,000 generations, with the first 2,500,000 discarded as burn-in. Post burn-in trees, branch lengths, and node ages were summarized in TreeAnnotator and displayed in FigTree v.1.4.3.

Lastly, since many variants were minimally divergent, and many were shared by multiple populations, we constructed statistical parsimony networks of all haplotypes for COI and for phased alleles of CAD and KKV using TCS version 1.2.1 ([59]). Networks were estimated using the 95% reconnection limit between haplotypes.

## Additional files

Additional file 1: All individual specimens sampled, localities, extracts. (XLSX 86 kb)

Additional file 2: All unique haplotypes/alleles (COI, CAD, KKV, and ITS2) and GenBank #s. (XLSX 53 kb)

Additional file 3: Combined data file for analysis in nexus format. (NEX 421 kb)

## Abbreviations

CAD: Carbamoyl-Phosphate Synthetase 2
COI: cytochrome oxidase I
ITS2: internal transcribed spacer 2
KKV: krotzkopf verkehrt
